# You stay, but I Hop: Host shifting near and far co‐dominated the evolution of *Enchenopa* treehoppers

**DOI:** 10.1002/ece3.3815

**Published:** 2018-01-15

**Authors:** Yu‐Hsun Hsu, Reginald B. Cocroft, Robert L. Snyder, Chung‐Ping Lin

**Affiliations:** ^1^ Department of Life Science National Taiwan Normal University Taipei Taiwan; ^2^ Division of Biological Sciences University of Missouri Columbia MO USA; ^3^ Present address: Department of Biology State University of New York College at Potsdam Potsdam NY USA

**Keywords:** co‐speciation, ecological speciation, host shifting, Membracidae, phylogenetic tracking, treehoppers

## Abstract

The importance and prevalence of phylogenetic tracking between hosts and dependent organisms caused by co‐evolution and shifting between closely related host species have been debated for decades. Most studies of phylogenetic tracking among phytophagous insects and their host plants have been limited to insects feeding on a narrow range of host species. However, narrow host ranges can confound phylogenetic tracking (phylogenetic tracking hypothesis) with host shifting between hosts of intermediate relationship (intermediate hypothesis). Here, we investigated the evolutionary history of the *Enchenopa binotata* complex of treehoppers. Each species in this complex has high host fidelity, but the entire complex uses hosts across eight plant orders. The phylogenies of *E. binotata* were reconstructed to evaluate whether (1) tracking host phylogeny; or (2) shifting between intermediately related host plants better explains the evolutionary history of *E. binotata*. Our results suggest that *E. binotata* primarily shifted between both distant and intermediate host plants regardless of host phylogeny and less frequently tracked the phylogeny of their hosts. These findings indicate that phytophagous insects with high host fidelity, such as *E. binotata*, are capable of adaptation not only to closely related host plants but also to novel hosts, likely with diverse phenology and defense mechanisms.

## 1 INTRODUCTION

Elucidating patterns of species richness and mechanisms of speciation are major goals in the study of ecology and evolution. Ecological speciation occurs when two taxa evolve reproductive isolation (i.e., barriers to gene flow) due to divergent selection between environments (Nosil, 2012; Schluter & Rambaut, 1996) and has been proposed to be a major speciation mechanism (Schluter, 2009). For host‐associated organisms, a change in host may result in novel environments for new adaptation. Changes in host can be caused by either divergence between hosts (i.e., co‐evolution) or a shift between hosts (i.e., host shifting; Page, 2003; Carmona, Fitzpatrick, & Johnson, 2015; Soudi, Reinhold, & Engqvist, 2015). The timing of host divergence is critical for the timing of divergence of dependent organisms in co‐evolution but not host shifting, but both mechanisms may result in phylogenetic tracking between hosts and their dependent organisms.

Two commonly considered concepts of phylogenetic tracking are Fahrenholz's parallel cladogenesis and Szidat's co‐phylogeny (Eichler, 1948). Parallel cladogenesis occurs when the evolution of parasites matches the evolution of their hosts (Eichler, 1948; Fahrenholz, 1913; Timm, 1983). Previous studies have shown that both co‐evolution and host shifting can result in parallel cladogenesis (Charleston & Robertson, 2002). By contrast, Szidat's co‐phylogenetic concept focuses on phylogenetic tracking caused by co‐evolution and postulates that ancestral hosts harbor more ancestral parasites (Eichler, 1948; Krasnov, Kiefer, Warburton, & Khokhlova, 2016; Szidat, 1939).

Although phytophagous insects have been a major focus in studies of host‐associated speciation (Antwi, Sword, & Medina, 2015; Ehrlich & Raven, 1964; Knolhoff & Heckel, 2014; Matsubayashi, Ohshima, & Nosil, 2010), phylogenetic tracking between phytophagous insects and their host plants has rarely been tested (Winkler & Mitter, 2008; also reviewed in de Vienne et al., 2013; Suchan & Alvarez, 2015). de Vienne et al. (2013) reviewed 86 studies reporting co‐phylogenetic analyses, of which only 12 examined phytophagous insects and their host plants. Similarly, only nine studies testing phylogenetic tracking between plants and insects were included in Suchan and Alvarez (2015)'s review. Both reviews concluded that there is a lack of support for phylogenetic tracking in insect–plant relationships.

However, in more than one‐third of the studies of insect–plant interactions reviewed, the insects feed on only one plant order (or even one genus in several cases; de Vienne et al., 2013; Suchan & Alvarez, 2015). Such a narrow host range makes it difficult to distinguish phylogenetic tracking from host shifting between hosts of intermediate similarity. Alternatively, Nyman (2010) argued that a novel host of high similarity (usually a sister taxon) will not generate the disruptive selection required for speciation and that an insect is unlikely to colonize a novel host with little similarity to the original host. The intermediate hypothesis posits that the maximum probability of insect speciation occurs when alternative hosts are of intermediate similarity in resource space, as determined by the resource that is critical to the fitness of the focal insect (Nyman, 2010). Such resources may include the secondary chemical compounds, nutritional content, or phenology of a plant, depending on the specific restrictions in each insect–plant interaction (Heard, 2012; Nyman, 2010). Comprehensive quantifications of the distance in resource space, however, are rare and not always applicable (Heard, 2012). Phylogenetically related plants often share similar physiological, morphological, and phenological characteristics due to phylogenetic conservatism (Cornwell et al., 2014; Davies et al., 2013; Liu et al., 2015). Therefore, the phylogenetic distance between host plants may represent the relative distance between host plants in the resource space to a certain degree.

**Figure 1:**
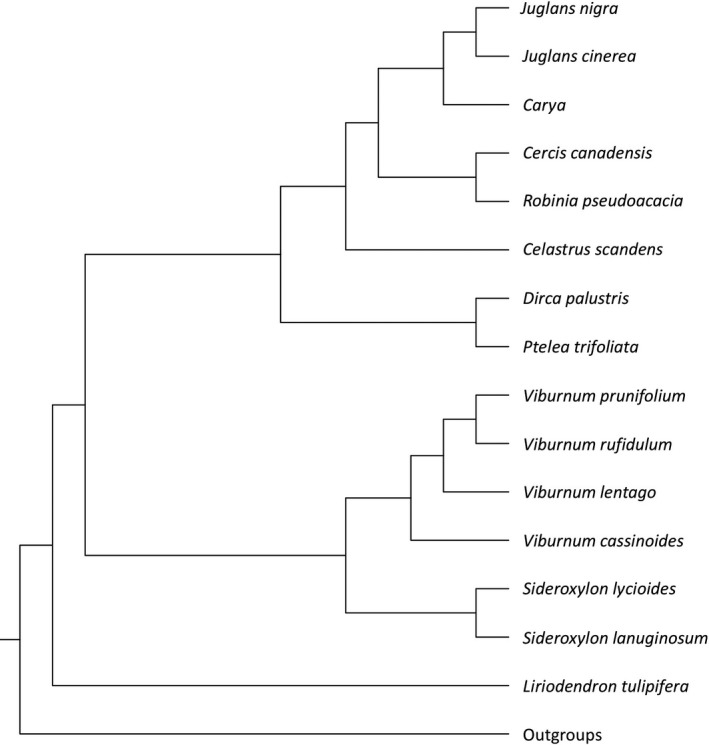
The phylogeny of the host plants of the *Enchenopa binotata* species complex (Modified from Winkworth & Donoghue, 2005; Aradhya et al., 2007; Manos et al., 2007; Soltis et al., 2011; Ruhfel et al., 2014)

The *Enchenopa binotata* species complex of treehoppers in Eastern North America is one of the best‐known examples of ecological speciation in phytophagous insects (Nosil, 2012; Wood, 1993). The host plants of *E. binotata* include eight plant orders (Wood, 1980; Wood & Guttman, 1982; Lin & Wood, 2002; Hamilton & Cocroft, 2009; Figure 1), with possible cases in another three plant orders (*Cornus* of Cornales, *Tilia* of Malvales, and *Ceanothus* of Rosales; Hamilton & Cocroft, 2009). The wide range of host plants for the *E. binotata* species complex provides a unique opportunity to test whether phylogenetic tracking or the intermediate hypothesis best explains insect–plant interactions.

It is not clear how interactions with host plants shaped the evolutionary history of the *E. binotata* species complex. Almost all *E. binotata* species are specialists, and each of them specialized in only one host plant species. The exceptions are *E. binotata* that feed on multiple species of *Viburnum* or *Carya*, but even these *E. binotata* are restricted to a single host genus (Lin & Wood, 2002). Many of the host plants used by the *E. binotata* species complex occur sympatrically, resulting in overlapping distributions of *E. binotata* species (Lin & Wood, 2002). The eggs of univoltine *E. binotata* species hatch asynchronously due to differences in water content among host plants in spring, but their subsequent life‐history stages are similar in duration (Wood, 1993; Wood & Guttman, 1982). The asynchronous first mating dates caused by the different hatching dates therefore result in assortative mating through temporal segregation (Wood & Keese, 1990). In the rare cases where adult *E. binotata* from different host plants meet, they tend to mate with conspecifics as a result of female preference for male vibrational mating signals (Rodriguez, Sullivan, & Cocroft, 2004; Rodriguez, Ramaswamy, & Cocroft, 2006; Cocroft, Rodríguez, & Hunt, 2008; Cocroft, Rodriguez, & Hunt, 2010; but see Rodriguez, Haen, Cocroft, & Fowler‐Finn, 2012 for a lack of male preference for signals of conspecific females).

In this study, we tested the concordance of the evolutionary histories of the *E. binotata* species complex and their host plants. The specific predictions derived from the phylogenetic tracking hypothesis were as follows. First, the phylogeny of the *E. binotata* species complex was predicted to match the host plant phylogeny (parallel cladogenesis). Second, more ancestral host plant species were predicted to harbor more ancestral *E. binotata* species (co‐phylogenesis). Alternatively, according to the intermediate hypothesis, host shifting in *E. binotata* was expected to occur more often between host plant species with intermediate distance in resource space. As the major reproductive barrier between *E. binotata* species is the difference in hatching dates caused by host plant phenology (Wood, 1980, 1993), which is phylogenetically conserved (Davies et al., 2013), we used the phylogenetic distance between host plants to represent the distance in resource space.

## 2 METHODS

### 2.1 Sample collection

A total of 61 *Enchenopa* individuals collected from across the species’ ranges were used for phylogenetic reconstruction (Table 1). Among the sampled specimens, 44 belonged to the *E. binotata* species complex and were collected from 15 host species of 10 genera from Eastern North America. We sampled the majority of *E. binotata* as 2nd–5th instar nymphs to permit accurate host association and species identification based on nymphal characteristics (Pratt & Wood, 1992). When nymphs were absent, we collected male adults and identified them by species‐specific mating signals (Cocroft et al., 2010). The remaining 17 *Enchenopa* species were collected from Central America and used as outgroups in phylogenetic analyses. Most outgroup specimens were collected as adults without host plant information. All specimens were preserved in 95% EtOH at −20 to −80°C for long‐term storage before DNA extraction.

**Table 1:** The collection locations of *Enchenopa* specimens, their associated host plants, and the GenBank accession numbers of their DNA sequences included in this study

No	Species	Host plant	Locality	Collectors	Collection date	Accession number (*CO1*)	Accession number (*EF1*α)
1	*E. binotata*	*Sideroxylon lycioides*	Moulton, AL	C.H. Dietrich	26‐May‐2004	KX791061	KX791122
2	*E. binotata*	*Sideroxylon lanuginosum*	Van Buren, MO	R.B. Cocroft	30‐Apr‐2006	KX791062	KX791123
3	*E. binotata*	*Sideroxylon lanuginosum*	Austin, TX	F.W. Stearns	01‐Apr‐2007	KX791063	KX791124
4	*E. binotata*	*Carya*	Mooresville, IN	R.L. Snyder	11‐Jun‐2004	KX791064	KX791125
5	*E. binotata*	*Carya*	Cloud Crossing, LA	C.P. Lin	27‐Apr‐2003	KX791065	KX791126
6	*E. binotata*	*Celastrus scandens*	Columbia, MO	R.B. Cocroft	01‐Jul‐2002	KX791066	KX791127
7	*E. binotata*	*Celastrus scandens*	Ithaca, NY	T.K. Wood & C.P. Lin	06‐Jul‐2002	KX791067	KX791128
8	*E. binotata*	*Celastrus scandens*	Stone Valley, PA	R.B. Cocroft & C.P. Lin	04‐Jun‐2003	KX791068	KX791129
9	*E. binotata*	*Cercis Canadensis*	Chinnabee, AL	C.P. Lin	01‐May‐2003	KX791069	KX791130
10	*E. binotata*	*Cercis Canadensis*	Rutledge, GA	R.B. Cocroft & C.P. Lin	07‐May‐2003	KX791070	KX791131
11	*E. binotata*	*Cercis Canadensis*	Effingham, IL	C.P. Lin	21‐May‐2003	KX791071	KX791132
12	*E. binotata*	*Cercis Canadensis*	Woodmont, MD	R.B. Cocroft & C.P. Lin	07‐Jun‐2003	KX791072	KX791133
13	*E. binotata*	*Cercis Canadensis*	Columbia, MO	R.B. Cocroft	01‐Jun‐2002	KX791073	KX791134
14	*E. binotata*	*Cercis Canadensis*	Tupelo, MS	C.P. Lin	29‐Apr‐2003	KX791074	KX791135
15	*E. binotata*	*Cercis Canadensis*	New Buffalo, PA	R.L. Snyder & N. Cai	09‐Jul‐2002	KX791075	KX791136
16	*E. binotata*	*Cercis Canadensis*	Parksville, TN	R.B. Cocroft & C.P. Lin	03‐May‐2003	KX791076	KX791137
17	*E. binotata*	*Cercis Canadensis*	Grafton, WV	R.B. Cocroft & C.P. Lin	06‐Jun‐2003	KX791077	KX791138
18	*E. binotata*	*Dirca palustris*	Bloomingdale, IN	R.E. Hunt	15‐Aug‐2004	KX791078	KX791139
19	*E. binotata*	*Dirca palustris*	White Lake, ON	R. Lee	01‐Jul‐2004	KX791079	KX791140
20	*Enchenopa* species	Unknown	Guatemala city, Guatemala	C.P. Lin	20‐Dec‐1999	KX791080	KX791141
21	*Enchenopa* species	Unknown	Gracias, Honduras	C.P. Lin & R.L. Snyder	22‐Jul‐2001	KX791081	KX791142
22	*Enchenopa* species	Unknown	Juticalpa, Honduras	C.P. Lin & R.L. Snyder	24‐Jul‐2001	KX791082	KX791143
23	*Enchenopa* species	Unknown	Lucerna, Honduras	C.P. Lin & R.L. Snyder	21‐Jul‐2001	KX791083	KX791144
24	*Enchenopa* species	Unknown	La Union, Honduras	C.P. Lin & R.L. Snyder	25‐Jul‐2001	KX791084	KX791145
25	*Enchenopa* species	Unknown	Bambito, Panama	T.K. Wood et al.	18‐Jan‐2000	KX791085	KX791146
26	*Enchenopa* species	Composite	Boquete, Panama	T.K. Wood & R.B. Cocroft	06‐Mar‐1998	KX791086	KX791147
27	*Enchenopa* species	Unknown	Panama	T.K. Wood et al.	01‐Jan‐2000	KX791087	KX791148
28	*Enchenopa* species	*Diphysa robinoides*	Pedasi, Panama	R.B. Cocroft	13‐Feb‐2000	KX791088	KX791149
29	*Enchenopa* species	Unknown	Gamboa, Panama	T.K. Wood et al.	21‐Jan‐2000	KX791089	KX791150
30	*Enchenopa* species	Unknown	La Union, Mexico	G. Moya Raygoza	24‐Oct‐2001	KX791090	KX791151
31	*Enchenopa* species	Unknown	La Huerta, Mexico	S.H. McKamey	16‐Oct‐2001	KX791091	KX791152
32	*Enchenopa* species	Unknown	Veracruz, Mexico	R.B. Cocroft & C.P. Lin	2007	KX791092	KX791153
33	*Enchenopa* species	Unknown	Veracruz, Mexico	R.B. Cocroft & C.P. Lin	2007	KX791093	KX791154
34	*Enchenopa* species	Unknown	Tegucigalpa, Honduras	C.P. Lin & R.L. Snyder	24‐Jul‐2001	KX791094	KX791155
35	*Enchenopa* species	Unknown	Trinidad, Honduras	C.P. Lin & R.L. Snyder	29‐Jul‐2001	KX791095	KX791156
36	*Enchenopa* species	Unknown	Chiriqui Grande, Panama	T.K. Wood & R.B. Cocroft	05‐Mar‐1998	KX791096	KX791157
37	*E. binotata*	*Juglans cinerea*	Bangor, NY	T.K. Wood	30‐Aug‐1997	KX791097	KX791158
38	*E. binotata*	*Juglans cinerea*	Ithaca, NY	T.K. Wood	16‐Jun‐1996	KX791098	KX791159
39	*E. binotata*	*Juglans nigra*	Woodmont, MD	R.B. Cocroft & C.P. Lin	07‐Jun‐2003	KX791099	KX791160
40	*E. binotata*	*Juglans nigra*	Columbia, MO	R.B. Cocroft	01‐Jul‐2002	KX791100	KX791161
41	*E. binotata*	*Liriodendron tulipifera*	Ithaca, NY	C.P. Lin	16‐Jul‐2002	KX791101	KX791162
42	*E. binotata*	*Liriodendron tulipifera*	Oxford, OH	R.L. Snyder	14‐Jun‐2004	KX791102	KX791163
43	*E. binotata*	*Liriodendron tulipifera*	Harveysburg, OH	R.L. Snyder	14‐Jun‐2004	KX791103	KX791164
44	*E. binotata*	*Ptelea trifoliata*	Yorkville, Il	R.L. Snyder	03‐Jun‐2004	KX791104	KX791165
45	*E. binotata*	*Ptelea trifoliata*	—	R.E. Hunt	01‐Jun‐2002	KX791105	KX791166
46	*E. binotata*	*Ptelea trifoliata*	Columbia, MO	R.B. Cocroft	01‐Jun‐2002	KX791106	KX791167
47	*E. binotata*	*Robinia pseudoacacia*	Woodmont, MD	R.B. Cocroft & C.P. Lin	07‐Jun‐2003	KX791107	KX791168
48	*E. binotata*	*Robinia pseudoacacia*	Columbia, MO	R.B. Cocroft	01‐Jul‐2002	KX791108	KX791169
49	*E. binotata*	*Robinia pseudoacacia*	Ithaca, NY	T.K. Wood & C.P. Lin	06‐Jul‐2002	KX791109	KX791170
50	*E. binotata*	*Robinia pseudoacacia*	Stone Valley, PA	R.B. Cocroft & C.P. Lin	04‐Jun‐2003	KX791110	KX791171
51	*E. binotata*	*Viburnum cassinoides*	Cherry Lane, NC	R.L. Snyder & N. Cai	05‐jun‐2003	KX791111	KX791172
52	*E. binotata*	*Viburnum cassinoides*	Davis, WV	R.B. Cocroft & C.P. Lin	06‐Jun‐2003	KX791112	KX791173
53	*E. binotata*	*Viburnum lentago*	Bernheim, KY	R.E. Hunt	01‐Jun‐2002	KX791113	KX791174
54	*E. binotata*	*Viburnum lentago*	Ridgeway, PA	R.B. Cocroft & C.P. Lin	05‐Jun‐2003	KX791114	KX791175
55	*E. binotata*	*Viburnum prunifolium*	Columbia, MO	R.B. Cocroft	27‐Jun‐2002	KX791115	KX791176
56	*E. binotata*	*Viburnum prunifolium*	Stone Valley, PA	R.B. Cocroft & C.P. Lin	04‐Jun‐2003	KX791116	KX791177
57	*E. binotata*	*Viburnum prunifolium*	Amherst, VA	R.L. Snyder & N. Cai	06‐Jun‐2003	KX791117	KX791178
58	*E. binotata*	*Viburnum rufidulum*	Rutledge, GA	R.B. Cocroft & C.P. Lin	07‐May‐2003	KX791118	KX791179
59	*E. binotata*	*Viburnum rufidulum*	Columbia, MO	R.B. Cocroft & C.P. Lin	01‐Jun‐2003	KX791119	KX791180
60	*E. binotata*	*Viburnum rufidulum*	Greenville, SC	C.P. Lin	08‐May‐2003	KX791120	KX791181
61	*E. binotata*	*Viburnum rufidulum*	Nashville, TN	R.B. Cocroft & C.P. Lin	02‐May‐2003	KX791121	KX791182

### 2.2 Molecular methods

For each sample, we extracted genomic DNA using a DNeasy animal tissue kit (Qiagen Inc., Valencia, CA, USA). We collected partial gene sequences from a nuclear intron of elongation factor 1 alpha (*EF1*α) and the mitochondrial cytochrome oxidase 1 (*CO1*) gene. We performed PCR amplification on an Eppendorf Mastercycler Gradient (Eppendorf North America, Westbury, NY, USA). To amplify *EF1*α, we used the following PCR primer set: For3 (mod) (5′GGTGACAACGTTGGTTTCAAC) and Cho8 (mod) (5′AATGTGAGCGGTGTGACAATC) (modified from Hillis, Moritz, & Mable, 1996). For *CO1*, the PCR primers Ron (C1‐J‐1751), Calvin (C1‐N‐2725), and Calvin1 (5′GTTGWGGRAARAAWGTTAARTTWACTCC) were used (Lin, Danforth, & Wood, 2004).

For each sample, the PCR contained ~50 ng of genomic DNA in a 30‐μl reaction with 0.1 μmol/L primer, 1.5 mmol/L MgCl_2_, 0.2 mmol/L each dNTP, and 1 unit of Tag DNA polymerase (GoTag, Promega Corp., Madison, WI, USA). The thermal cycling conditions for each primer set were as follows: 35 cycles of denaturation at 94**°**C for 50 s, annealing at 52**°**C (*EF1*α) or 50**°**C (*CO1*) for 1 min, and extension at 72**°**C for 1 min, with a final step at 72**°**C for 6 min. We used Sequencer v4.5 (Gene Codes Corp., Ann Arbor, MI, USA) to edit and align the resulting sequences.

The Sanger sequencing method was not compatible with some PCR products of the *EF1*α gene, and therefore, we cloned these PCR amplicons using a TOPO^®^ Ta cloning kit (Invitrogen, Life Technologies Corp., Carlsbad, CA, USA) before sequencing. We isolated the plasmid DNA using a PureLink Quick Plasmid Mini Purification kit (Invitrogen, Life Technologies Corp., Carlsbad, CA, USA) and sequenced five colonies for each cloning reaction. We sequenced the inserted region of the vector using the universal primers supplied in the kit.

### 2.3 Phylogenetic reconstruction of *Enchenopa binotata*

We first reconstructed the gene trees of *CO1* and *EF1*α separately using the parameters of the substitution models described below. Both gene trees supported the clustering of *E. binotata* into two clades, and those feeding on *Cercis* and *Liriodendron* were sister groups (Figure S1). However, most tree branches had low support, suggesting that the phylogenetic information from each of the two genes alone was insufficient to resolve the relationships. We therefore combined *CO1* (875 bp) and *EF1*α (870 bp) using a supermatrix approach in Sequence Matrix v1.8 (Vaidya, Lohman, & Meier, 2011). For maximum likelihood (ML) and Bayesian phylogenetic analyses, the best‐fit nucleotide substitution model was selected in jModelTest v0.1.1 (Posada, 2008) using the Bayesian Information Criterion (BIC). The *CO1* sequences were partitioned by each codon position, whereas the *EF1*α sequences were partitioned as introns and exons. ML trees were obtained using RAxML v8.2.X (Stamatakis, 2014) under the GTRCATI model, followed by 10,000 bootstrap replicates to estimate the 95% credible intervals (95% CIs). Bayesian analyses were performed using MrBayes v3.2.6 (Ronquist et al., 2012) under the best‐fit model for each partition (i.e., GTR for the 1st and 3rd codon positions in *CO1*, JC69 for the 2nd codon position in *CO1* and exons in *EF1*α, and HKY for the introns in *EF1*α). In the Markov Chain Monte Carlo (MCMC) process, we ran four chains with 5 × 10^7^ generations. Convergence of the MCMC process was diagnosed when the average standard deviation of the split frequencies was 0. The first 25% of the MCMC samples were discarded as burn‐in.

We reconstructed the species trees and estimated divergence times using BEAST v1.8.2 (Drummond, Suchard, Xie, & Rambaut, 2012) and the best‐fit model for each partition (i.e., TN93 + I + G with three partitions for *CO1*, TN93 with two partitions for exons in *EF1*α, and HKY with no codon partitions for introns in *EF1*α). To estimate divergence times, we fit the lognormal relaxed clock (uncorrelated) with a range of mutation rates of *CO1* in insects between the standard and revised rates (1.15 × 10^−8^ and 1.77 × 10^−8^, respectively, with an average of 1.46 × 10^−8^ mutations/site/year; Brower & DeSalle, 1998; Papadopoulou, Anastasiou, & Vogler, 2010). For exons and introns in *EF1*α, we applied a range of mutation rates between the highest and lowest mutation rates reported in insects (exons: 0.2942 × 10^−8^ and 0.558 × 10^−8^, respectively, with an average of 0.426 × 10^−8^ mutations/site/year; introns: 0.732 × 10^−8^ and 2.27 × 10^−8^, respectively, with an average of 1.501 × 10^−8^ mutations/site/year; reviewed in Lin & Danforth, 2004). For the MCMC settings, we ran three independent chains with the same set of parameters, each for 1 × 10^8^ generations. The convergence of each MCMC chain was diagnosed by the effective sample sizes of the parameters (ESS > 200). The results of these three chains were combined using LogCombiner v1.8.2 (Drummond et al., 2012), with the first 2.5 × 10^7^ MCMC samples of each chain discarded as burn‐in.

### 2.4 Phylogeny of host plants

We extracted the tree topology of the eight plant orders that cover the confirmed hosts of the *E. binotata* species complex (i.e., Celastrales, Dipsacales, Ericales, Fabales, Fagales, Magnoliales, Malvales, and Sapindales) according to the backbone of angiosperm phylogenies from Soltis et al. (2011) and Ruhfel, Gitzendanner, Soltis, Soltis, and Burleigh (2014). We then detangled the subtrees within the two orders with multiple branches: Fagales and Dipsacales. Three host taxa were included in the order Fagales: *Juglans nigra*,* J. cinerea*, and *Carya*. According to molecular and morphological data, *J. nigra* and *J. cinerea* are more closely related to each other than to *Carya* (Aradhya, Potter, Gao, & Simon, 2007; Manos et al., 2007). Among the four host species of Dipsacales, *Viburnum prunifolium,* and *V. rufidulum* are the most closely related species, followed by *V. lentago* and *V. cassinoides* (Winkworth & Donoghue, 2005). The final host plant phylogeny (Figure 1) included only host plants of the collected *E. binotata* in this study and encompassed nearly all host plant species that have been recorded more than once.

### 2.5 Evaluating the major causes of speciation in *E. binotata*

We conducted an event‐based parallel cladogenesis reconstruction analysis in *Jane* version 4 (Conow, Fielder, Ovadia, & Libeskind‐Hadas, 2010) to evaluate whether the *E. binotata* phylogeny matches the angiosperm phylogeny. The high level of host fidelity in *E. binotata* together with differences in life‐history timing caused by host phenology (Wood, 1980; Wood & Keese, 1990; Wood, Tilmon, Shantz, Harris, & Pesek, 1999) suggest that host shifting might be more costly than phylogenetic tracking with the original host species. We therefore set the cost of phylogenetic tracking at a lower level (0 units) and the cost of host shifting at three different levels (0, 1, and 2 units) to explore the sensitivity of the results to various weighting schemes. The costs of duplication, loss, and failure to diverge were set as the default settings (all equal to 1, with the exception of duplication cost = 0 when the cost of host shifting = 0 due to the software limitation). We ran each simulation in the solve mode of *Jane* with 500 iterations; 1,000 different solutions were considered at each iteration according to the suggestions of Conow et al. (2010). We also ran the randomization analyses with random tip mapping (Conow, 2013; Conow et al., 2010) and 1,000 steps with the same setting to evaluate the robustness of the results. We conducted the same set of analyses with host‐only plant phylogeny, and the results remain the same. Therefore, we only present results based on the angiosperm phylogeny.

To test for co‐phylogenesis between host plants and *E. binotata*, we used a phylogenetic generalized linear mixed model (GLMM). Because divergence times were not available for all host plants, we used the clade rank (i.e., the number of speciation events between the basal nodes of a phylogenetic tree and a given taxon; Knouft & Page, 2003) as a proxy for species age. To include as much information in the plant phylogeny as possible, we counted the clade rank of host plants by considering all nodes of the order‐level angiosperm tree reconstructed by Ruhfel et al. (2014). Conducting the same analysis by counting the clade rank of host plants in a host‐only phylogeny produced similar results, thus we presented only the results based on the order‐level tree because information on all orders of the angiosperm tree was included.

We fit a Poisson GLMM (Poisson error with log‐link function) with MCMC methods in R version 3.3.0 (R Core Team 2013) using the package *MCMCglmm* (Hadfield, 2010). We fit the *E. binotata* clade rank as the response, the clade rank of the host plants as the fixed effect, and the host phylogenetic information as the random effect. The host genus was also fit as a random effect to manage the unbalanced sampling across the host genera. We calculated the phylogenetic heritability, *H* ^2^, as the phylogenetic variance (Lynch, 1991), equivalent to Pagel's λ (i.e., a measure of the tendency of related species to resemble one another; Pagel, 1999; Hansen & Orzack, 2005; Hadfield & Nakagawa, 2010). To test the robustness of our results, we ran the same analysis with host plant clade ranks calculated from the host‐only plant phylogeny, and the results agreed with the main analysis (Table S1). We therefore only present the main analysis in the main text.

We fit the *MCMCglmm* default priors for fixed effects, an inverse Wishart prior for random effects, and residuals as *V *=* *1 and ν* *= 0.02, where *V* was defined as the variance and ν as the degree of belief in *V*. We ran each model for 5 × 10^6^ iterations, followed by another 5 × 10^6^ iterations and a thinning interval of 500. We ran three parallel chains for both models and conducted Gelman‐Rubin diagnostics to check for convergence (Gelman & Rubin, 1992). For each model, we report the means of the posterior distributions and their 95% CIs as the parameter estimates.

## 3 RESULTS

### 3.1 *Enchenopa* Phylogenetic trees

**Figure 2:**
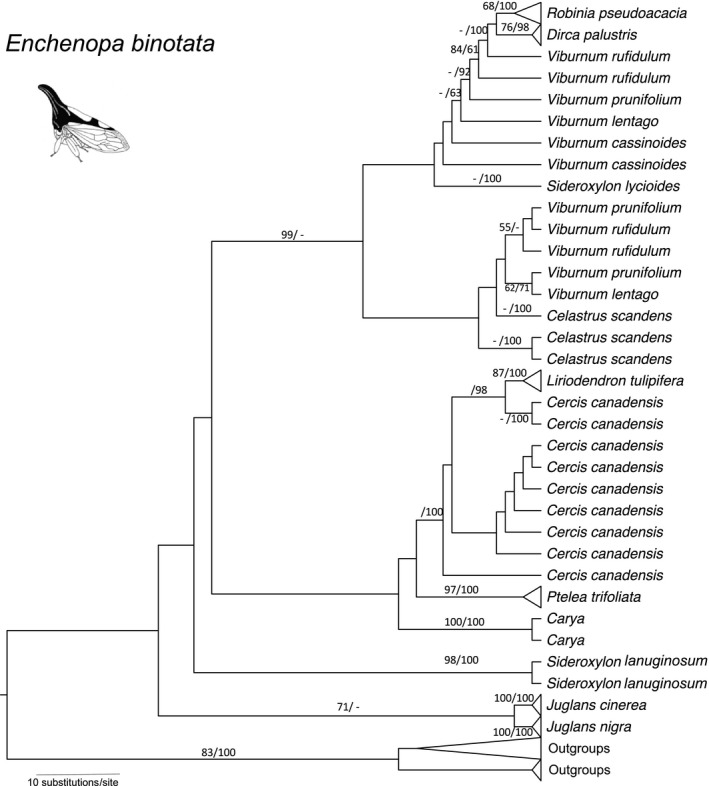
Maximum‐likelihood gene tree of *Enchenopa binotata* based on the GTR model. The numbers above the branches are bootstrap values of 100 replicates (%)/Bayesian posterior probability (%; bootstrap values and Bayesian posterior probability <50% not shown)

**Figure 3:**
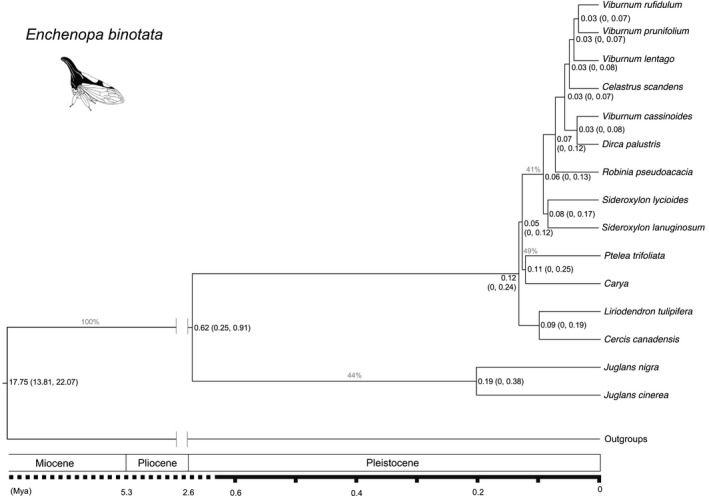
The Bayesian species tree of *Enchenopa binotata* from the software BEAST v1.8.2. The number at each node indicates the estimated divergence time with their 95% credible intervals, whereas the number above each branch presents the Bayesian posterior probability (BPP <40% not shown)

The combined sequence matrix used for phylogenetic reconstruction was 1645 bp (GenBank accession numbers: *CO1*, KX791061‐KX791121; *EF1*α, KX791122‐KX791182; Table 1), with 429 parsimony‐informative sites. The phylogenetic trees indicated that among *E. binotata* species feeding on 10 different host plant genera, six were monophyletic (*Carya*,* Dirca*,* Juglans, Liriodendron, Ptelea,* and *Robinia*), with the first five having more than 70% branch support from both ML bootstrapping and Bayesian posterior probability (BPP) (Figure 2). For the remaining *E. binotata*, those feeding on *Celastrus* and *Cercis* were paraphyletic, and those feeding on *Viburnum* and *Sideroxylon* were polyphyletic, with various levels of branch support (from <50% to 100% BPP), suggesting a probable effect of incomplete lineage sorting in recently diverged species. The support values of the *E. binotata* species tree were generally low (BPP mostly <30%; Figure 3), raising doubts on the robustness of the phylogeny of *E. binotata* and thus the evolutionary history interpreted from it. However, this tree topology largely agrees with a previous *E. binotata* phylogeny reconstructed from mtDNA with limited geographical sampling (Lin & Wood, 2002), indicating congruence of different data sets.

Because the topologies of the reconstructed phylogenetic trees based on different sets of mutation rates were similar, we only present the phylogenetic tree reconstructed with the average mutation rates (Figure 3). According to this phylogenetic tree, the Eastern North American *E. binotata* complex was estimated to have diverged from Central American *Enchenopa* species approximately 17.7 million years ago (Mya) in the early Miocene (Figure 3). The *E. binotata* feeding on *Juglans* diverged from the rest of *E. binotata* ~0.62 Mya in the middle Pleistocene, with a BPP of 100%. The remaining *E. binotata* species were inferred to diverge from each other more recently, between 29 and 117 thousand years ago in the late Pleistocene.

### 3.2 Evaluating the major causes of speciation in *E. binotata*

**Figure 4:**
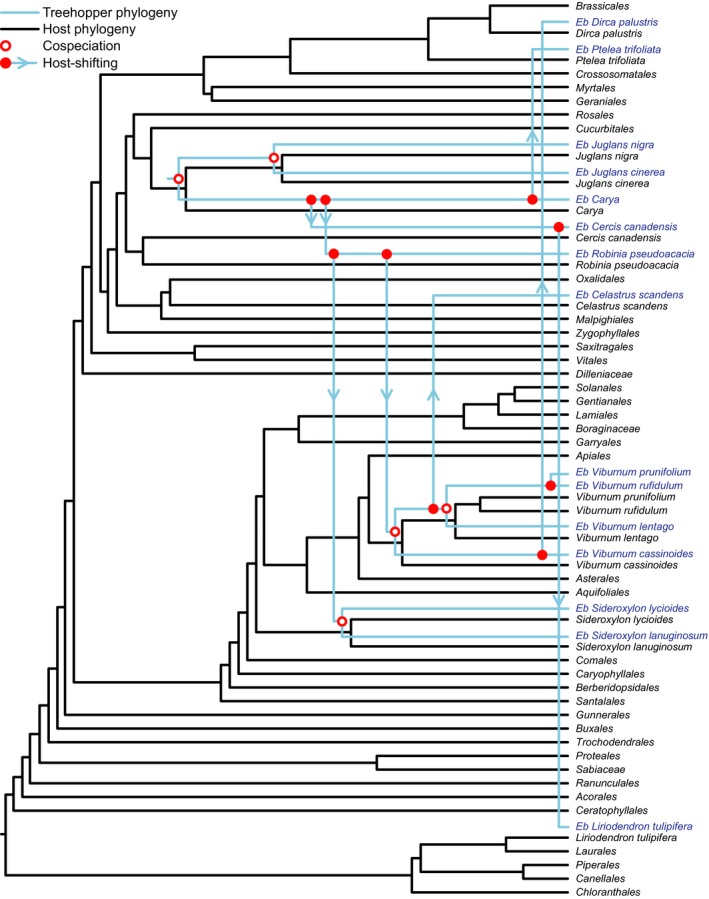
The host‐shifting and co‐evolution history of *Enchenopa binotata* and their host plants estimated from the event‐based parallel cladogenesis reconstruction analysis

The three different cost combinations of phylogenetic tracking and host shifting all resulted in more host shifting than phylogenetic tracking (Table 2). Therefore, we only present detailed results for the moderate setting, in which the cost of host shifting was 1. A total of 100,000 solutions were obtained from the event‐based parallel cladogenesis analyses. All solutions suggested that the *E. binotata* complex and their host plants were estimated to undergo more host‐shifting events (*n *=* *8) than phylogenetic‐tracking events (*n *=* *5; Figure 4). The randomization analysis reported that the cost combinations from our results were significantly better than random cost combinations (*p *<* *.01). In addition, the estimated host shifting occurred regardless of the host plant relationships in the phylogenetic tree (Figure 4), indicating that host shifting was not restricted to plant species with intermediate relationships.

**Table 2:** The estimated number of phylogenetic‐tracking and host‐shifting events in the evolutionary history of the *Enchenopa binotata* complex according to event‐based analyses

Cost settings (Phylogenetic tracking, host shifting)	Estimated number of phylogenetic‐tracking events	Estimated number of host‐shifting events
(0, 0)	1	13
(0, 1)	5	8
(0, 2)	5	8

According to the test of co‐phylogenesis, the clade ranks of the host plant orders were not significantly associated with the clade ranks of *E. binotata* (posterior mean = 0.06, 95% CI = −0.05 to 0.18). The estimated posterior mean of the phylogenetic heritability (*H* ^2^) was 50.37%, indicating a significant phylogenetic signal for the distribution of speciation events in the phylogeny.

## 4 DISCUSSION

We found that the degree of phylogenetic association between the *Enchenopa binotata* species complex and their host plants does not support phylogenetic tracking as the major mechanism of speciation of *E. binotata*. In contrast, our results suggested that host shifting dominated the evolutionary history of the *E. binotata* complex. However, the existence of a few estimated events indicates that phylogenetic tracking might have occurred, although at a much lower frequency than host shifting. These results indicate that the plant–insect evolutionary interactions may have been the product of multiple mechanisms. Furthermore, because the host shifting between *E. binotata* species was not limited to intermediately related host plants but also occurred across several plant orders (e.g., shifting from *Cercis* in Fabales to *Liriodendron* in Magnoliales), our results are also inconsistent with the intermediate hypothesis of host shifting.

Of the two causes of phylogenetic tracking, co‐evolution has been the focus of extensive attention for decades (reviewed in Suchan & Alvarez, 2015). Vigorous discussions of co‐evolution in recent years (Althoff, Segraves, & Johnson, 2014; Carmona et al., 2015; Hembry, Yoder, & Goodman, 2014; Martínez‐Aquino, 2016; Suchan & Alvarez, 2015; Thompson, 2014) have suggested that event‐based analyses may be inadequate because even if co‐evolution dominated the evolutionary history of host plants and associated insects, their phylogenies may still be incongruent due to other events (e.g., occasional host shifting, lineage duplications, and/or lineage extinction; de Vienne et al., 2013). It is therefore advisable to determine the age of each node in the host and insect phylogenies to establish a more reliable test of temporal congruence in co‐evolution events (Hafner et al., 1994; Page, 1996). This method cannot be formally applied in the current study due to the lack of divergence times among the host plants. However, the relatively newly evolved host plant genera (e.g., *Juglans* and *Carya*) were estimated to have diverged more than 20 Mya in the early Neogene (Xiang et al., 2014), which is a time frame much earlier than the divergence of the *E. binotata* feeding on these plants (~0.1 to 0.16 Mya). This incongruence of divergence times between the *E. binotata* species and their host plants further indicates that phylogenetic tracking, especially co‐evolution, is not the dominant force of divergence in this species complex.

Our results instead suggest that host shifting between distantly related host plants plays a dominant role in the divergence of the *E. binotata* species complex. This conclusion is not surprising given that current evidence of co‐evolution mostly comes from parasites or symbionts living inside their hosts (e.g., endosymbionts or ectosymbionts on internal surfaces of hosts; reviewed in de Vienne et al., 2013). In contrast to parasites and symbionts, *E. binotata* treehoppers are mobile and can freely move between plants, providing ample opportunities to encounter novel hosts.

The secondary chemical compounds, nutritional content, and phenology of plants (i.e., the contents of the resource space for each host plant) often constrain their utilization by insects and thus are frequently associated with insect divergence (especially the phenology of host plants in the divergence of *E. binotata*; Wood & Keese, 1990; Bruce, 2015). Closely related plant species frequently share similarities in chemical compounds, nutrition, and phenology (Davies et al., 2013; Prasad et al., 2012). Therefore, host shifting is traditionally expected to occur more often between closely related host plants. Given the lack of support in this study for phylogenetic tracking, this is unlikely to be the case between *E. binotata* and their host plants. Alternatively, the intermediate hypothesis predicts that the maximum probability of host shifting in *E. binotata* occurs between intermediately related host plants because these plants tend to share intermediate similarity with each other in the resource space. However, this hypothesis is not supported because the results suggest that *E. binotata* shifts between both intermediately related species (e.g., *Viburnum* in Dipsacales and *Dirca* in Malvales; Figures 1 and 4) and distantly related ones (e.g., *Carya* in Fagales and *Liriodendron* in Magnoliales). These results suggest that distance in resource space does not explain the patterns of host shifting in *E. binotata*. As a phytophagous insect with high host fidelity (Wood et al., 1999), it is fascinating that *E. binotata* remains sufficiently evolutionarily flexible to shift between distant host plant species regardless of host phylogeny or positions in the resource space. These results also indicate that although plant phenology is largely responsible for the divergence between *E. binotata*, these treehoppers exhibit a range of plasticity to overcome potentially strong host selection, adapt to temporal differences between novel host plants, and successfully shift between distant host plants (Wood, 1993; Wood et al., 1999).

Once the restrictions of host plant use are relaxed, the temporal and spatial distributions of host plants and insects may become major determinants of host shifting (de Vienne et al., 2013). Specifically, the timing of the overlapping distribution of *E. binotata* species might be a key factor in host‐shifting events. The recent overlapping area between *Carya* and *Juglans* is larger than that between *Carya* and *Ptelea*, similar to the distributions of *E. binotata* feeding on these plants (reviewed in Lin & Wood, 2002). Therefore, if the present distribution reflects past range to some extent, host shifting between *Carya* and *Juglans* is expected to be more likely than that between *Carya* and *Ptelea*. However, our results indicate that the recent range shifts of host plants and *E. binotata* are insufficient to explain host shifting by *E. binotata*. Alternatively, *Enchenopa* might have shifted to a novel host when these plant species expanded their distributions in Eastern North America after the last glacial period (~26,000 to 19,000 years ago; Clark et al., 2009). According to available data on the paleo‐distribution of *Carya* and *Juglans*, both were distributed in Eastern North America ~20,000 years ago and reached 20% of dominance in at least half of this area 10,000–12,000 years ago (Delcourt & Delcourt, 1987). However, the *E. binotata* feeding on these plants are estimated to have diverged much earlier (114,000 and 189,000 years ago, respectively; Figure 3), which does not support this potential explanation. More detailed analyses, such as ecological niche modeling of both *Enchenopa* and their host plants, are required to further evaluate whether the paleo‐distribution of the host plants was associated with host shifting of the *E. binotata* complex, which may have facilitated the speciation of the latter.

In conclusion, we have demonstrated that both phylogenetic tracking and host shifting played roles in the evolutionary history of the *E. binotata* species complex, with greater importance of host shifting compared to phylogenetic tracking. These results suggest multiple modes of insect–plant evolutionary interactions. Furthermore, *Enchenopa* treehoppers are capable of shifting between distantly related host plants, which is inconsistent with the general assumption of host shifting. This capability indicates that the evolution of *E. binotata* with high host fidelity could surprisingly be relaxed from the constraints set by the phenologies and defense mechanisms of their host plants. Such evolutionary plasticity suggests that, in addition to the dependence of host choice on host characteristics, recent and historical changes in the distributions of host plants and *Enchenopa* are key factors shaping the host‐shifting history of *E. binotata*. Testing these hypotheses will require more detailed information on the distributions of both *Enchenopa* and their host plants across different time frames.

## CONFLICT OF INTEREST

None declared.

## AUTHOR CONTRIBUTIONS

CPL and RBC designed the study; CPL, RBC, and RLS collected samples; CPL and RLS extracted DNA and conducted sequencing; YHH conducted phylogenetic and statistical analyses; YHH wrote the draft with input from CPL and RBC.
