# Supplementary material for: You stay, but I Hop: Host shifting near and far co‐dominated the evolution of Enchenopa treehoppers
Source: Ecol Evol. 2018 Jan 15;8(4):1954–65. doi: 10.1002/ece3.3815 (PMC5817127; doi:10.1002/ece3.3815)
Supplement: Supplementary file 1 [file ECE3-8-1954-s001.docx]

B)

ML best tree based on *ef1α*

A)

ML best tree based on CO1

Figure S1. The ML best tree of A) *CO1* gene tree and B) *ef1α* gene tree.

Table S1. The results of phylogenetic generalized linear mixed model based on event-base analysis testing for cophylogenesis between host-plants and *Enchenopa binotata*. Results are the mean(95% credible interval) based on the host plant clade ranks calculated from A) the angiosperm phylogeny, and B) the host-only plant phylogeny.

|  | | **A** | **B** |
| --- | --- | --- | --- |
| Fixed effects | |  |  |
|  | Intercept | 0.33 (-2.53 to 3.21) | 1.86 (0.77 to 2.74) |
|  | Host plant clade rank | 0.06 (-0.05 to 0.18) | 0.01 (-0.20 to 0.19) |
| Random effects | |  |  |
|  | Host phylogenetic information | 0.17 (0 to 0.70) | 0.003 (0 to 0.34) |
|  | Host plant genus | 0.05 (0 to 0.19) | 0.01 (0 to 0.23) |
| Dispersion | | 0.02 (0 to 0.07) | 0.002 (0 to 0.08) |
